# Advancing collagen‐related pathology assessment through second‐harmonic generation imaging

**DOI:** 10.1111/jmi.70098

**Published:** 2026-04-23

**Authors:** Jackson Rodrigues, Manikanth Karnati, Gagan Raju, Edwin Pious, Ankur Gogoi, Guan‐Yu Zhuo, Nirmal Mazumder, Fu‐Jen Kao

**Affiliations:** ^1^ Institute of Biophotonics National Yang Ming Chiao Tung University Taipei Taiwan; ^2^ Department of Biophysics Manipal School of Life Sciences Manipal Academy of Higher Education Manipal India; ^3^ Department of Physics Jagannath Barooah University Jorhat Assam India

**Keywords:** artificial intelligence, collagen, microscopy, polarisation

## Abstract

Collagen remodelling and dysregulation are the hallmarks of diverse pathological conditions. In this context, second‐harmonic generation (SHG) imaging has emerged as a powerful label‐free modality for assessing collagen. This offers submicron resolution, intrinsic optical sectioning, and deeper imaging capabilities without the need for exogenous agents. Additionally, polarisation‐resolved SHG (P‐SHG) further enhances orientation‐sensitive information on collagen architecture. This enables the elucidation of subtle structural alterations that are often invisible to conventional imaging techniques. This nonlinear technique has demonstrated utility in diagnosing diseases, particularly in assessing tumour progression, where collagen remodelling is correlated with disease severity and prognosis. Furthermore, quantitative image analysis, using metrics such as fibre density, orientation, anisotropy, and alignment, provides objective measures of collagen remodelling. P‐SHG complements these analyses by enabling the retrieval of molecular susceptibility ratios (e.g., χ33/χ31), degrees of polarisation (DOLP), and orientation distribution functions, yielding more profound insights into the fibrillar ultrastructure and molecular organisation. These quantitative descriptors are being increasingly integrated into clinically relevant feature extraction methods. The incorporation of these features in artificial intelligence (AI) methods has enhanced SHG‐based pathological assessment. AI‐driven models can automatically classify tissues, detect pathological patterns, and correlate the collagen microstructure with clinical outcomes, addressing the bottleneck of manual interpretation and enhancing reproducibility. Future perspectives highlight the integration of SHG and P‐SHG with deep‐tissue multiphoton imaging, accompanied by explainable AI/ML‐driven quantification, which will improve diagnostic precision, reproducibility, and clinical adoption. Ultimately, the convergence of optical innovation, deep learning, and translational engineering will lead to the establishment of collagen pathology assessment through SHG and P‐SHG as reliable, accessible tools for diverse clinical applications.

## 1 INTRODUCTION

Collagen, the most abundant structural protein in the human body, forms the fundamental framework of connective tissues and underpins their mechanical integrity. Organising into hierarchical fibrillar networks provides the strength and architecture of the skin, bone, cartilage, vasculature, and other extracellular matrices.^1^, ^2^, ^3^, ^4^, ^5^ In addition to its structural role, collagen is actively involved in dynamic biological processes such as tissue morphogenesis, remodelling, wound healing, and pathological progression.^6^, ^7^, ^8^, ^9^ Structural alterations not only act as diagnostic and prognostic biomarkers but also contribute mechanistically to disease pathogenesis.^10^, ^11^ Consequently, precise visualisation and quantitative characterisation of collagen architecture are pivotal for advancing collagen research. Collagen has traditionally been examined using methods such as histological staining for tissue‐wide distribution, electron and atomic force microscopy for ultrastructural and mechanical characterisation, and vibrational spectroscopy for chemical composition determination.^12^, ^13^, ^14^, ^15^, ^16^, ^17^ Histopathology provides accessible and widely adopted assessments of collagen; however, it is constrained by staining variability, observer‐dependent interpretation, and its largely qualitative nature. Over the past few decades, multiphoton fluorescence microscopy has emerged as a complementary optical tool, offering deep tissue penetration and three‐dimensional resolution. However, it typically requires exogenous dyes or labels, is susceptible to photobleaching, and can perturb tissue physiology.^18^, ^19^, ^20^, ^21^ These cumulative limitations underscore the need for a label‐free, quantitative, and clinically adaptable imaging modality capable of capturing the collagen architecture with both high resolution and depth.

In this context, second‐harmonic generation (SHG) microscopy has become an essential modality for collagen characterisation.^22^, ^23^, ^24^ SHG microscopy is a nonlinear imaging method in which two photons interact with noncentrosymmetric structures such as fibrillar collagen to produce light at half the excitation wavelength, yielding label‐free contrast. Many biological structures, including fibrillar collagen, microtubules, and myosin, fulfil this noncentrosymmetric requirement. Ultrafast lasers are essential for inducing nonlinear interactions, such as SHG, which requires extremely high peak intensities achievable through femtosecond or picosecond pulse trains; details about the theoretical framework are discussed in the Supplementary Information. This nonlinear property enables quantitative visualisation of collagen organisation with submicron resolution and penetration depths suitable for both ex vivo and in vivo applications, thereby overcoming the critical limitations of histological, spectroscopic, and electron‐based methods. Compared with the conventional approaches summarised in Table 1, SHG provides several decisive advantages. It is inherently label‐free, highly specific for fibrillar collagen, and allows deep tissue penetration with three‐dimensional resolution in intact samples.^25^, ^26^, ^27^, ^28^, ^29^, ^30^, ^31^

**TABLE 1:** Overview of widely used approaches for collagen characterisation and their limitations compared with those of SHG microscopy.^32^, ^33^, ^34^, ^35^, ^36^

Method	Principal features	Limitations	Relative advantages of SHG/P‐SHG microscopy
Histological staining	Widely used; highlights collagen distribution; compatible with routine pathology	Requires fixation and staining; subjectivity in interpretation; semiquantitative	SHG is label‐free, provides intrinsic contrast, and yields quantitative, reproducible metrics; P‐SHG further reveals fibril orientation and anisotropy
Electron microscopy (EM)	Nanometre resolution; ultrastructural detail	Destructive sample prep; ex vivo only; small fields of view	SHG allows 3D imaging in intact tissues with larger fields of view and in situ capability as compared to EM/AFM; P‐SHG allows for orientation mapping
Atomic force microscopy (AFM)	Measures nanoscale stiffness and collagen mechanics	Labour‐intensive; small sampling area; surface‐limited imaging	SHG enables rapid volumetric imaging of collagen microarchitecture; P‐SHG adds molecular‐level information on alignment and, in some cases, discrimination of collagen subtypes
Raman/FTIR spectroscopy	Chemical specificity; detects crosslinks and composition	Weak signals; slow acquisition; limited spatial resolution	SHG provides higher spatial resolution and faster imaging while being nondestructive as compared to Raman FTIR; P‐SHG contributes anisotropy and crosslink‐sensitive metrics
Multiphoton fluorescence microscopy	High‐resolution 3D imaging with exogenous labels	Requires dyes/labels; photobleaching; potential tissue alteration	SHG generates intrinsic contrast without labels, avoiding artefacts and phototoxicity; P‐SHG enables label‐free probing of collagen orientation and structural heterogeneity

SHG and polarisation‐resolved SHG (P‐SHG) microscopy have gained significant importance in biomedical research because they enable the direct visualisation of collagen alterations that correlate with tumour invasiveness, fibrosis severity, and connective tissue disorganisation. Derived biomarkers such as fibre alignment, density, anisotropy, and texture serve as objective indicators of disease progression and therapeutic response. Quantitative image analysis further enhances this potential by converting SHG datasets into reproducible metrics through algorithms that extract orientation distributions, anisotropy indices, and texture descriptors, thereby reducing observer bias and improving cross‐study comparability. More recently, artificial intelligence (AI) approaches, including machine learning and deep learning, have enabled the automated classification of diseases, prediction of outcomes, and detection of subtle remodelling features that exceed human perception.

This review highlights recent advances by examining the principles of SHG and P‐SHG microscopy, their applications in collagen pathology, and the emerging integration of AI.

## 2 ASSESSMENT OF COLLAGEN PATHOLOGY BY SHG MICROSCOPY

SHG and P‐SHG microscopy thus serve as powerful label‐free tools for assessing collagen pathology, which is essential for understanding disease progression.^37^, ^38^ During disease progression, collagen undergoes distinct reorganisations; thus, quantitative assessment of collagen architecture is vital for understanding tissue function. Imaging parameters in SHG and P‐SHG microscopy are crucial for assessing collagen pathology with high specificity and sensitivity. Both the forward‐to‐backward (*F*/*B*) ratio, which indicates fibril thickness and packing, and the SHG intensity, which reflects collagen density, are critical metrics.^39^ P‐SHG improves structural insights via polarisation‐based measures. The anisotropy parameter (*r*), computed from parallel and perpendicular SHG intensities, quantifies the degree of fibre alignment. In contrast, Stokes‐vector analysis provides the degree of linear polarisation (DOLP), the degree of circular polarisation (DOCP), and the overall degree of polarisation (DOP), which reveal molecular orientation and chirality. Nonlinear susceptibility tensor element ratios (e.g., χ_33_/χ_31_) offer insights into the molecular structure of collagen.^40^ Collagen acts as an important biomarker for studying various conditions, such as cancer, fibrosis, wound healing, and regenerative medicine.^41^

### 2.1 Cancer

In a study by Golaraei et al., P‐SHG imaging demonstrated distinct differences in collagen ultrastructure between the breast cancer stroma and normal tissue. The *R* values of malignant tissues were greater (2.21 for triple‐positive vs. 2.10 for normal), indicating that collagen rearrangement occurred. Orientation dispersion decreased (straighter fibres), and DLP decreased (e.g., 0.69 in double‐positive vs. 0.80 in normal), indicating increased fibre disorder and depolarisation.^42^ In invasive breast cancer (IBC‐ST), SHG demonstrated uniformly increased peritumoural collagen metrics across all subtypes, suggesting a shared mechanism of tumour margin remodelling. The quantity, uniformity, and organisation of intratumoural collagen differ significantly by subtype. SHG imaging indicated that neoadjuvant chemotherapy (NACT) causes subtype‐specific collagen remodelling in breast cancers. In HER2+ and triple‐negative breast cancer (TNBC) tumours, the forward‐to‐backward SHG ratio (*F*/*B*) increased dramatically in the tumour core following NACT, indicating altered fibre packing, although the tumour–stroma interface was unaltered. In TNBC, orientation dispersion decreased, indicating greater fibre alignment, a phenomenon not observed in HER2+ tumours, as shown in Figure 1I.^43^ P‐SHG analysis of breast cancer tissues revealed different collagen ultrastructures in the perilesional and tumour stroma. Perilesional collagen exhibited a high supramolecular symmetry parameter (*S* = 0.51), indicating a trigonal structure, whereas the tumour stroma consisted of aligned, cylindrical fibres with poor symmetry (*S* = 0.16). Symmetry mapping (*S*) outperformed anisotropy (*R*) in differentiating regions (*p* < 0.001), allowing for submicron resolution regardless of fibril size or concentration.^44^ These P‐SHG‐derived metrics serve as label‐free, quantitative indicators for distinguishing between benign and malignant tissue and for classifying tumours on the basis of collagen organisation and integrity, as shown in Figure 1II.^45^ A practical example from a recent breast cancer stromal study began with ImageJ for batch intensity normalisation and preliminary orientation maps, then transitioned to MATLAB for P‐SHG anisotropy fitting and finally utilised Python to integrate all the metrics into a machine learning classifier. This modular approach is valuable for clinical translation.^46^ In ovarian cancer, SHG microscopy of fallopian tube tissues demonstrated that compared with normal and precursor lesions, high‐grade serous ovarian cancer (HGSOC) has much greater collagen fibre alignment and straightness. P‐SHG of ovarian tissues revealed substantial differences in collagen ultrastructure among normal stroma, benign tumours and HGS malignancies (stage III and stage IV tumours), as shown in Figure 1III. Collagen levels peak in stage III HGS, indicating desmoplastic remodelling. The α‐helical pitch angles of benign tumours were the greatest, whereas those of HGS tissues were lower, indicating reduced Col III. SHG anisotropy and circular dichroism are greatest in normal stroma and lowest in malignant stroma, indicating altered fibrillar order and chirality.^47^

**FIGURE 1:**
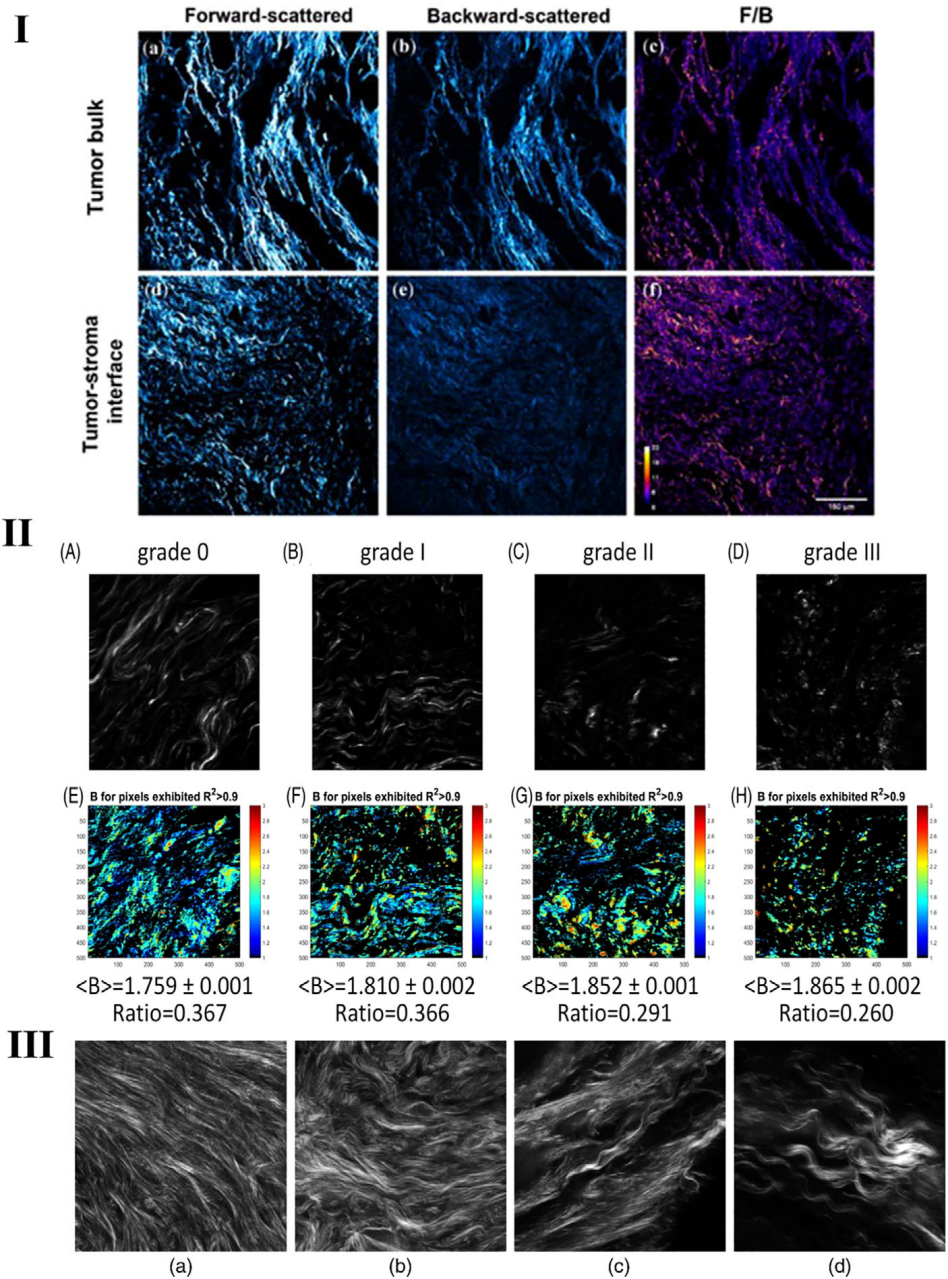
(I) SHG imaging was carried out on the tumour mass.^43^ (II) Representative images of different grades of breast tissues and the results of the FFT analysis of each sample shown in A–D.^45^ (III) SHG images of ovarian tissue classification: (a) normal, (b) benign tumour, (c) stage III, and (d) stage IV.^47^ All the figures are reproduced with kind permission from the journal.

### 2.2 Fibrosis

SHG microscopy of liver biopsies from nonalcoholic fatty liver disease (NAFLD) and nonalcoholic steatohepatitis (NASH) patients detected collagen changes earlier and more precisely than conventional histology did. The SHG intensity and high‐intensity pixel area increased as the disease progressed, and forward‐to‐backward SHG ratios, which represent fibre shape and packing, increased dramatically with fibrosis progression, distinguishing between early and advanced NASH, as shown in Figure 2I.^48^ In another study by Ng et al., SHG microscopy of a mouse NASH model demonstrated dynamic, region‐specific collagen remodelling during fibrosis progression and reversal. Mice fed a high‐fat sugar–water diet presented a 137% increase in the collagen proportionate area (CPA), particularly in Zone 2, whereas diet reversal reduced the CPA by 22%. The fibrosis score and 71 SHG‐derived fibrillar markers were used to monitor illness development and regression, as shown in Figure 2II. SHG also revealed early perisinusoidal fibres and advanced septal fibrosis.^49^ P‐SHG analysis of liver fibrosis revealed that the dispersion of the anisotropy parameter (ρ) varies according to the collagen architecture: tendons have low ρ dispersion because of straight, ordered fibrils; skin has moderate dispersion from supercoiled fibrils with slight disorder; and liver vessels have high ρ dispersion from variable supercoil angles. In the fibrotic liver, ρ dispersion decreases, indicating increased fibrillar alignment and stiffness.^50^

**FIGURE 2:**
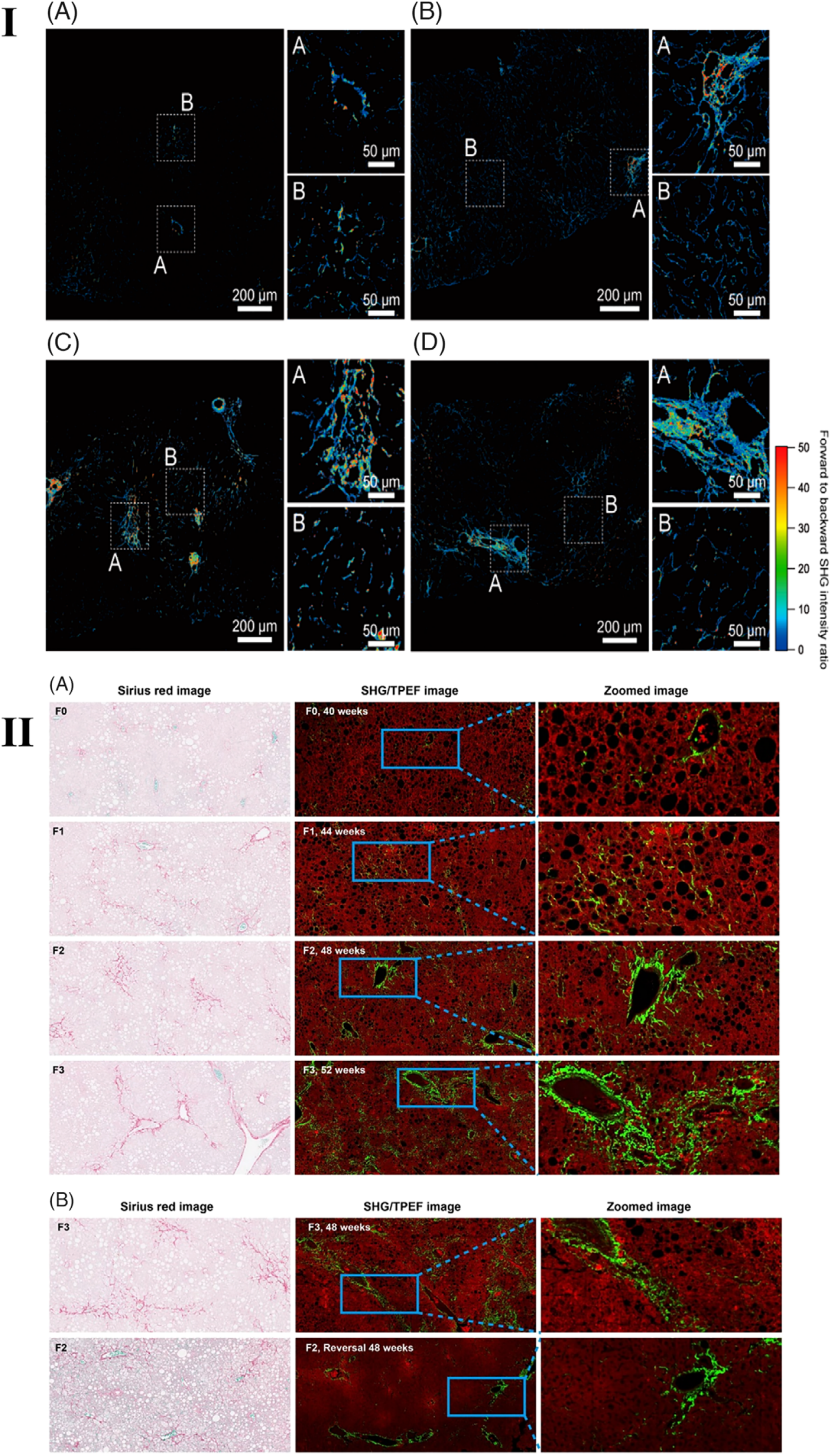
(I) SHG intensity ratio images of the livers of (A) the ultra‐early stage of NAFLD, (B) the ultra‐early stage of NAFLD, (C) the early stage of NASH, (D) the advanced stage of NASH.^48^ (II) SHG images of mouse liver sections at various time points during (A) fibrosis progression and (B) fibrosis regression, fibrous collagen (green) and liver parenchyma (red).^49^ All the figures are reproduced with kind permission from the journal.

### 2.3 Bone and cartilage

In bone and cartilage pathology, SHG microscopy along with optical scattering and Monte Carlo modelling revealed zone‐specific collagen degradation patterns in enzymatically treated cartilage, as shown in Figure 3. Trypsin caused only minor superficial alterations, whereas collagenase significantly altered deep‐zone collagen, lowering scattering anisotropy (*g*). SHG‐derived texture and fibre metrics enabled a classification accuracy of 89%–97% across treatment groups. *F*/*B* and heterogeneity revealed enzyme‐specific fibril disruption, with collagenase having the lowest *F*/*B* values.^51^ In a study by Mingalone et al., SHG imaging of early osteoarthritic cartilage revealed collagen loss and rearrangement following joint destabilisation. The *F*/*B* ratios decreased across all zones, indicating fibril thinning, but the directional variance increased in the superficial zone but decreased in the radial zone, indicating zone‐specific fibre disarray and alignment. In a study by Makkithaya et al., P‐SHG was used to detect molecular and structural alterations in collagen to distinguish among healthy, OA, and rheumatoid arthritis (RA) cartilage. OA had a greater tensor ratio (χ33/χ31) and DOLP, indicating a shift to type I collagen and improved fibrillar organisation. RA demonstrated intermediate modifications with a more disorganised orientation. Texture analysis revealed unique remodelling patterns in OA and deterioration in RA, confirming that P‐SHG is a label‐free technique for evaluating cartilage illness.^44^ P‐SHG imaging of bovine and human articular cartilage revealed zone‐specific collagen organisation compatible with Benninghoff's hypothesis. The deep zone had the highest fibril order (*I*_2_). Under tensile strain, the collagen fibrils aligned and increased in a specific order on the basis of the initial orientation. P‐SHG revealed diverse regions of fibril organisation in OA cartilage, indicating regional degradation.^52^

**FIGURE 3:**
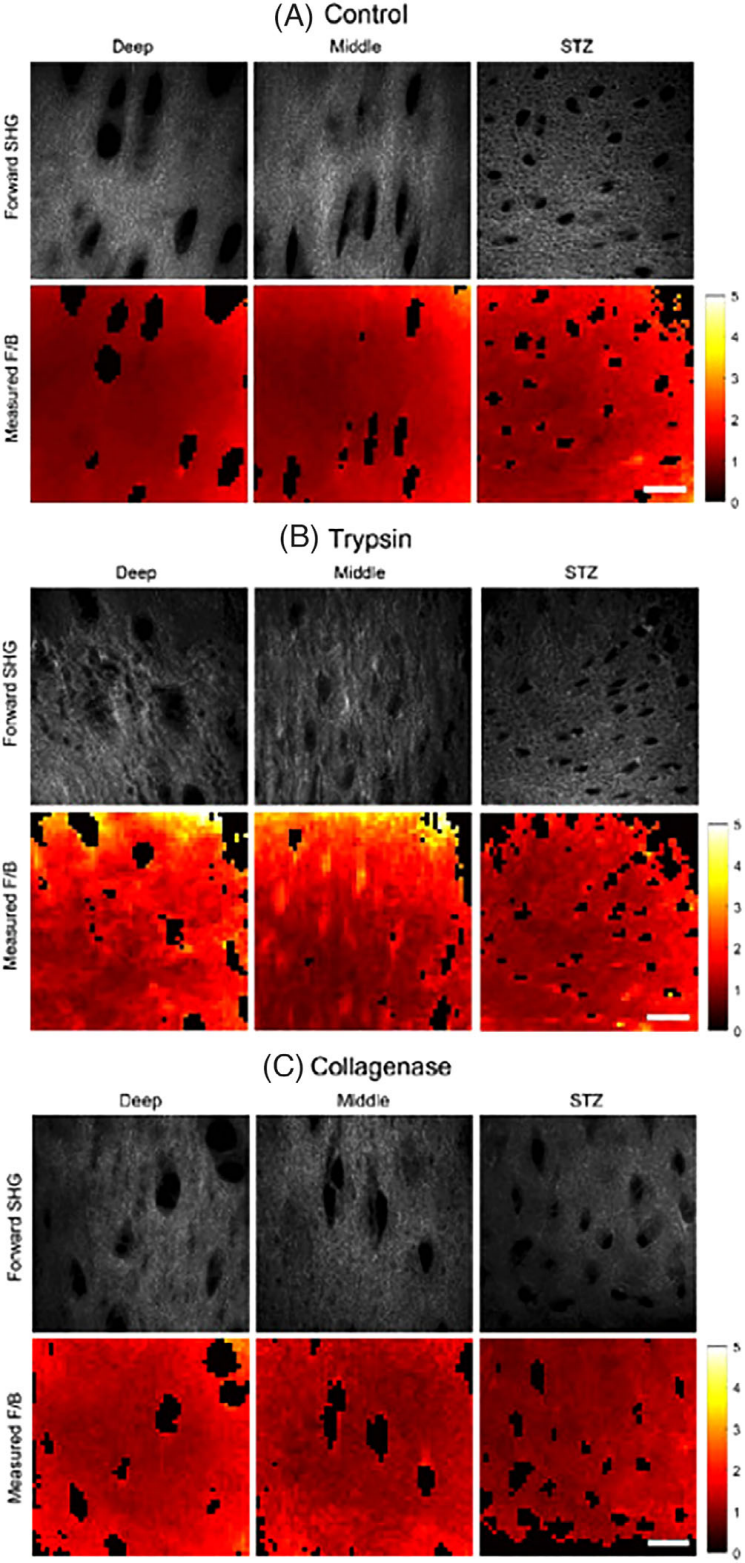
SHG images of (A) normal, (B) trypsin‐degraded, and (C) collagenase‐degraded cartilage.^51^ All the figures are reproduced with kind permission from the journal.

**FIGURE 4:**
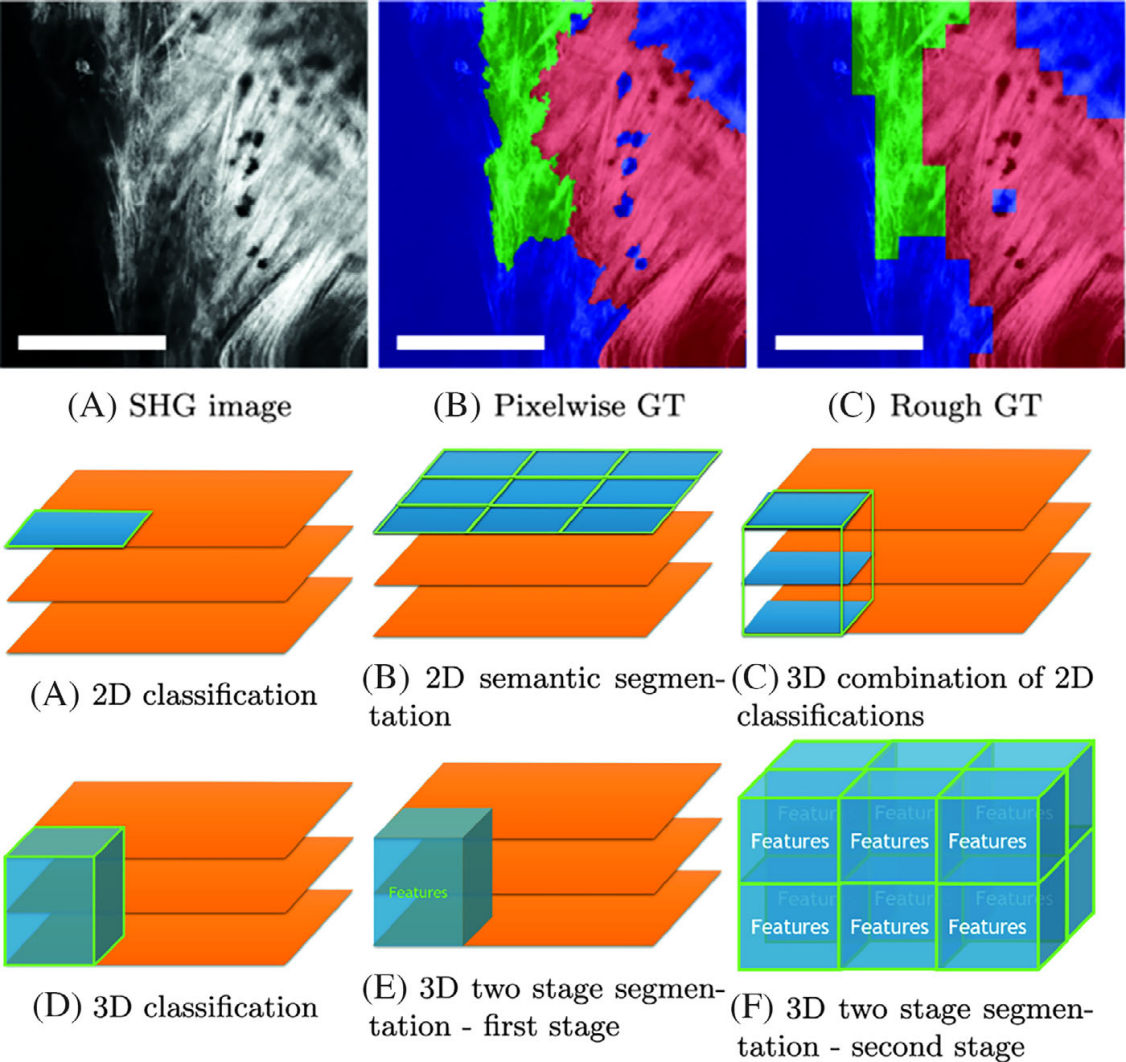
Representation of the SHG input image with ground‐truth segmentation showing uncertain borders (top). Schematic illustration of the input–output formats for different segmentation methods (bottom). The figure is adopted from Ref. (69).

## 3 QUANTITATIVE IMAGE ANALYSIS OF THE SHG MICROSCOPY

Quantitative analysis transforms the complex visual patterns in SHG images into objective numerical parameters that pathologists can rely on. In practical workflows, both researchers and clinicians focus on a core set of descriptors that collectively depict collagen architecture, including intensity‐based statistics, collagen content (expressed as area fraction or density), fibre orientation, anisotropy or degree of alignment, and texture measures reflecting heterogeneity. Together, these parameters create a quantitative ‘fingerprint’ of the ECM status, which is often strongly correlated with disease stage or clinical outcome.

For SHG image analysis, two broad methods are routinely applied to determine the orientation of collagen fibres. The first involves Fourier domain analysis, where the image is transformed into a spatial frequency map. The angular distribution of energy in this frequency space indicates prevailing fibre orientations, a strategy widely used in ocular connective tissues and other collagen‐rich organs to quantify preferred alignment patterns. The second approach relies on structure tensor (gradient‐based) analysis, which estimates local fibre directionality within each pixel neighbourhood. These local orientation vectors can be combined to generate full‐field orientation maps and dispersion indices. The widely adopted *OrientationJ* plugin is a typical implementation, with extensive validation in vascular and soft tissue studies. Polarisation‐resolved SHG (P‐SHG) provides an additional dimension of detail. By systematically rotating the excitation polarisation and modelling the resulting intensity modulation, P‐SHG can distinguish overlapping collagen bundles and determine their orientations with greater specificity, which is especially valuable in densely packed stromal regions.

Anisotropy complements orientation by quantifying the degree to which fibres share the same direction. In image space, this can be expressed as *coherency* (from the structure tensor) or as the eccentricity/magnitude of the Fourier angular spectrum, both of which offer robust measures of alignment. High anisotropy values indicate ordered collagen organisation, whereas low values indicate isotropy or structural disorganisation. At the molecular bundle level, P‐SHG offers anisotropy measures such as the ρ (rho) ratio or related susceptibility‐derived contrasts, which reveal differences in fibrillar packing and tilt parameters that may change with disease progression or mechanical loading. Texture features further describe microarchitectural patterns in the tissue. Measures derived from the grey‐level co‐occurrence matrix (GLCM), including contrast, homogeneity, correlation, and entropy, quantify the fluctuations in pixel intensities across various spatial relations. These parameters are beneficial for distinguishing between normal and remodelled stroma when traditional metrics such as density or mean intensity fail to capture subtle changes.

Crucially, these collagen metrics work together to provide a richer, multidimensional understanding of tissue pathology. Recognising the complementary nature of these metrics, most clinical and translational research now reports a concise panel of quantitative features. This panel typically includes (i) collagen density or area fraction to quantify deposition, (ii) preferred fibre orientation along with a dispersion index to capture directional variability, (iii) an anisotropy or coherency score measuring the degree of fibre alignment, and (iv) 2–4 texture features derived from a grey‐level co‐occurrence matrix or related analyses that characterise microarchitectural patterns and heterogeneity. Together, this multiparametric approach offers a more comprehensive and robust assessment of collagen remodelling across a range of pathological conditions, improving the accuracy and predictive value of SHG and P‐SHG imaging in clinical contexts.^53^, ^54^

### 3.1 Correlative quantitative SHG/P‐SHG analysis with histopathology and other modalities

SHG integrated into digital pathology platforms has emerged as a powerful approach for assessing fibrosis. The translational significance of SHG and P‐SHG lies in their ability to produce label‐free, quantitative collagen metrics that align with established clinical gold standards, such as haematoxylin & eosin (H&E), picrosirius red under polarisation (PSR‐POL), fibrosis staging systems, such as METAVIR and Ishak, and complementary modalities, including two‐photon excited fluorescence (TPEF), wide‐field polarisation microscopy, and biomechanical measurements. Across multiple organ systems, these correlations reinforce the use of SHG/P‐SHG as a sensitive companion to conventional histopathology, enhancing objectivity while preserving clinical interpretability. In liver fibrosis, the multimodal SHG/TPEF ‘qFibrosis’ pipeline quantifies the collagen burden and architecture in unstained biopsies and strongly aligns with the METAVIR and Ishak scores; moreover, subtle longitudinal changes during antiviral therapy changes are often missed by semiquantitative pathologist scoring. Similar results were obtained when SHG was compared with PSR‐POL: Although PSR‐POL may yield higher apparent fibre counts or lengths because of dye‐specific birefringence effects, both methods agree on overall trends in collagen abundance and organisation.^54^ The complementary physical contrast, nonlinear scattering for SHG versus dye‐induced birefringence for PSR‐POL, means that reporting both can aid cross‐validation and smooth the adoption of SHG in clinical settings already anchored to PSR‐POL.

Early artificial intelligence approaches for analysing SHG images mainly relied on handcrafted feature extraction methods to describe collagen fibre morphology and texture.^55^, ^56^ These texture‐based features quantify collagen fibre size, length, anisotropy, and orientation and are combined with traditional classifiers such as support vector machines to distinguish normal tissue from pathological tissue, as seen in fibrosis and asthma assessments. While these feature‐based approaches provide valuable biological insights, they require substantial domain expertise for crafting meaningful descriptors and are limited in their ability to handle complex 3D collagen architectures.^57^, ^58^ These foundational works established quantitative metrics and benchmarked AI capabilities such that deep learning methods were later surpassed by automating feature extraction directly from raw SHG data, enhancing accuracy and generalisability. The contribution of SHG and P‐SHG imaging with AI is rapidly evolving in biomedical imaging. The advent of CNNs, a way to extract multiscale spatial patterns from SHG datasets, opened the door to integrate the spatial and polarisation‐dependent features in a single pipeline.

### 3.2 Overview: from proof‐of‐concept to clinical validation

The integration of AI with SHG imaging has evolved from early proof‐of‐concept studies to applications on clinically derived samples, demonstrating its translational adoption. Accurate prediction of second‐harmonic generation coefficients using graph neural networks, where crystal structures are represented as graphs and processed by graph neural networks to predict SHG coefficients. By encoding atoms as nodes and interatomic relations as edges, the model leverages convolutional messages to learn complex chemical–geometric interactions underlying nonlinear optical responses. This framework circumvents the high computational cost of density functional theory (DFT) calculations, enabling rapid, high‐throughput screening of candidate nonlinear optical materials with accuracy comparable to that of traditional methods.^59^ or automating viability assessment of chondrocytes in articular cartilage with deep learning–enhanced SHG and autofluorescence microscopy. Parallel efforts in pulmonary research have used texture‐based feature extraction and supervised learning to classify collagen remodelling in asthmatic versus control airways with high accuracy, establishing SHG‐AI pipelines as powerful tools for extracellular matrix quantification.^60^ More recently, clinical cartilage samples have been analysed with deep learning models such as YOLOv8 to automatically detect and quantify microcracks within complex SHG datasets.^61^ This progression from theoretical and preclinical demonstrations to clinically relevant tissue analysis highlights a clear improvement: AI‐augmented SHG is maturing into a reproducible, high‐throughput, and clinically translatable methodology for quantifying structural remodelling in health and disease.

When integrated with AI pipelines, the performance becomes twofold in regard to time savings: rapid image acquisition combined with automated classification or preannotation of regions of interest. AI‐assisted workflows in digital pathology have already demonstrated reductions in task‐specific reporting time and pathologist workload, with some models providing near‐instantaneous reporting once digital slides are available.^62^, ^63^ Importantly, the role of SHG should be framed as a complementary tool rather than a replacement for conventional histopathology. SHG coupled with AI has the potential to reduce workflow time for specific clinical use cases, such as intraoperative margin assessment or ex vivo tissue characterisation, but rigorous validation in real‐world, multicentre clinical workflows is still needed.^64^

### 3.3 AI architectures and feature strategies

In the literature on AI‐assisted SHG and P‐SHG image analysis, three broad methodological strategies have been reported. The first is physics‐informed feature extraction integrated with ML (spGLCM combined with random forest or XGBoost and GLCM with PCA followed by k‐means clustering).^65^ This approach has high interpretability even with smaller datasets and robustness against overfitting. It might fail to capture complex patterns of higher order that lie beyond handcrafted descriptors. The second strategy involves end‐to‐end deep learning architectures such as ResNet, InceptionV3, GoogleNet, and other CNN‐based models that operate without explicit feature engineering.^66^ These models perform automatic feature extraction and can adapt to heterogeneous datasets. The third is the hybrid framework that integrates physics‐informed features extracted from deep learning classifiers or regressors.^59^ This approach offers a balance between interpretability and predictive performance.

### 3.4 Trends in P‐SHG‐specific AI analysis

Polarisation‐resolved SHG offers a unique advantage in AI analysis: the data inherently encode directional and molecular‐order information about collagen fibres. AI models that explicitly exploit this, by using polarisation‐resolved texture descriptors or by training on angle‐resolved SHG stacks, tend to outperform those trained on single‐orientation SHG images. Angle‐resolved deep learning frameworks in polarisation imaging treat the polarisation information captured at multiple angles (e.g., 0°, 45°, 90°, and 135°) as additional input channels. This approach allows CNNs to directly learn polarisation‐dependent contrast patterns, enhancing their ability to analyse images by leveraging polarisation cues by incorporating the polarisation dimension as extra channels.^67^ This approach has been demonstrated in studies such as those using the PolaRGB dataset for reflection removal, where CNNs leverage the polarisation channels as complementary inputs to RGB for enhanced imaging performance.^68^

### 3.5 Collagen segmentation strategies

Assessment of the accurate pathology of the biological sample is achieved by harnessing the structural information from the collagen structure obtained via SHG imaging via segmentation of the collagen. In this context, deep learning algorithms have demonstrated their utility. CNNs consistently outperform conventional methods by providing accuracies above 90% even at greater imaging depths, thereby demonstrating the robustness of the delineation of collagen fibres across heterogeneous tissue environments.^63^ By utilising weights from pretrained 2D models, state‐of‐the‐art neural architectures (Inception‐ResNet‐3D) enable the 3D segmentation of local fibre orientations (Figure 4). This has demonstrated a scalable framework for resolving uncertain collagen alignments in volumetric SHG datasets.^69^

**FIGURE 5:**
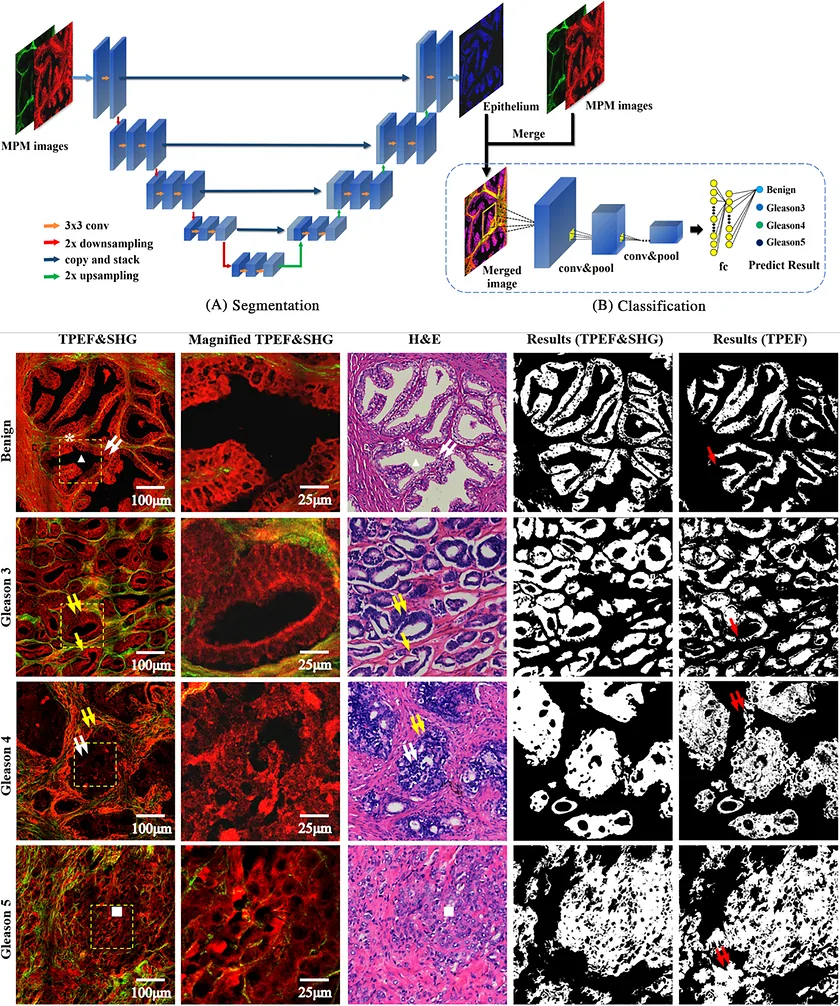
Label‐free multiphoton microscopy (MPM) combined with deep learning enables epithelial segmentation and automated Gleason grading in prostate cancer diagnosis (top). Representative TPEF&SHG images, along with corresponding H&E reference and segmentation results, illustrate the differentiation of benign tissue and prostate cancer Gleason patterns 3–5 (bottom). Key structures are marked: epithelium (white arrows), gland unit (yellow arrows), lumen (white triangle), fibromuscular tissue (asterisk), tumour nest (white rectangle), and segmentation errors (red arrows). Adopted from Ref. (70).

Segmentation has also been implemented in epithelium segmentation involving SHG from the collagen content in the tissue for the automated Gleason grading of prostate cancer via label‐free multiphoton microscopy combined with deep learning (Figure 5). Here, a U‐Net model achieved a pixel accuracy of 92.3% and a mean *F*1 score of 0.839 for epithelium segmentation, which was subsequently integrated into a classification pipeline for cancer grading.^70^ Furthermore, segmentation based on SHG imaging has been employed for the detection of structural damage to cartilage, where YOLOv8 was adapted to identify microcracks in the collagen network of articular cartilage. These microstructural lesions, which are detectable only with high‐resolution SHG, are clinically significant, as they can propagate under cyclic loading and initiate degenerative conditions such as osteoarthritis.^61^ Collectively, advances in segmentation by deep learning are enabling increasingly precise, high‐throughput analyses of collagen organisation, with direct implications for both diagnostic accuracy and mechanistic understanding of tissue pathology.

**FIGURE 6:**
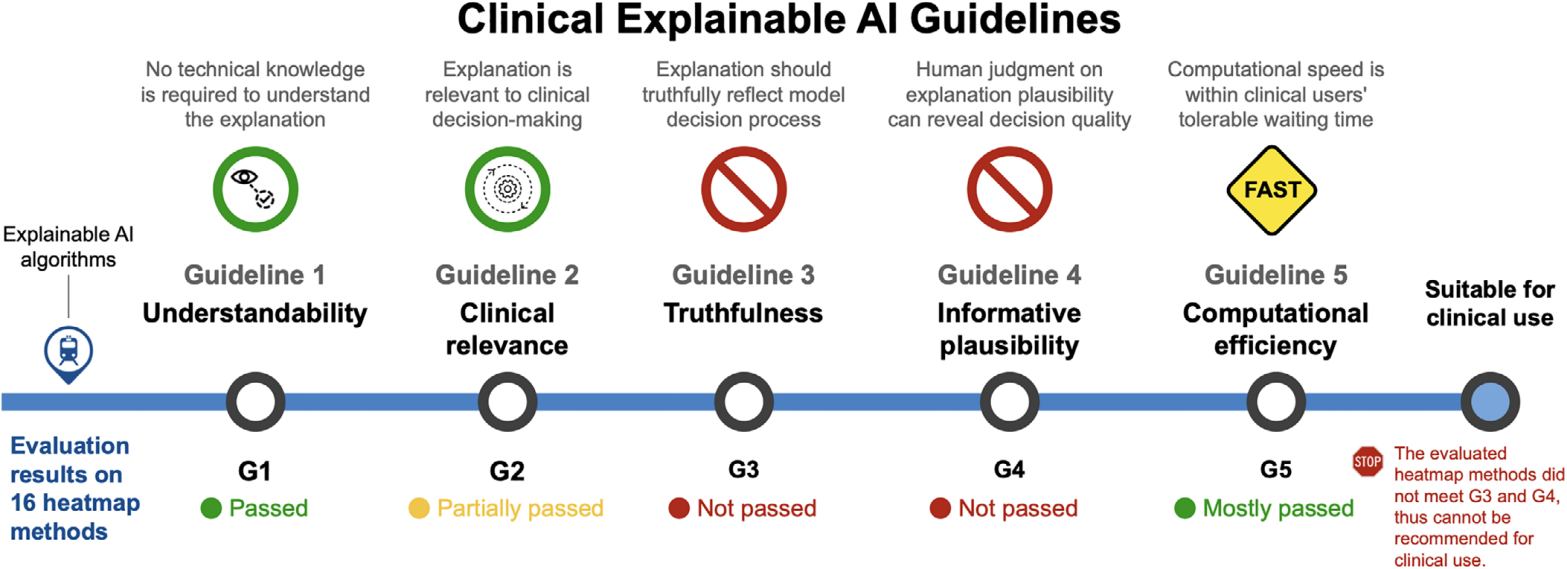
Clinical explainable AI guidelines. Explainable AI algorithms should meet the five criteria in the guidelines to be suitable for clinical use. The evaluation results for the 16 heatmap methods regarding the guideline criteria are shown at the bottom. Figure adopted from Ref. (76).

## 4 CHALLENGES AND LIMITATIONS OF COLLAGEN PATHOLOGY ASSESSMENT THROUGH SHG IMAGING

The application of SHG and P‐SHG imaging in collagen pathology assessment is associated with many practical implementation challenges. This limits the clinical application of this technique in routine clinical workflows. This technique relies on precise, high‐cost instrumentation that is required to obtain optimal image quality. This dependency on expertise and instrumentation can hinder accessibility in low‐resource settings. Another limitation of this technique is that its penetration depth is achievable with SHG. The method is robust in providing intrinsic optical sectioning and can image several hundred micrometres of depth in the tissue. However, the scattering and absorption effects on biological samples significantly degrade the signal at deeper optical planes. This is particularly affected in the case of backward collection of signals in in vivo imaging.^71^, ^72^ The molecular specificity of SHG is also narrow. As the technique is primarily sensitive to noncentrosymmetric structures, this characteristic is most commonly found in collagen and a few other proteins.^73^ Additionally, many SHG analysis workflows remain labour intensive and rely heavily on pixel‐by‐pixel computations and manual efforts. This not only prolongs data processing but also introduces the potential for user‐dependent bias and variability.^74^ Emerging deep learning pipelines offer promising solutions by automating feature extraction and quantification, thereby reducing subjectivity and accelerating analysis; however, widespread adoption will require large, standardised datasets and robust validation across different imaging systems. Despite promising pilot and proof‐of‐concept studies on SHG and related nonlinear optical imaging using AI, several clinical translation challenges remain. The dataset scarcity and generalisability are the key concerns of the application of AI in clinical implementation. Many studies use small, single‐centre cohorts under controlled imaging conditions; thus, the performance of these methods in diverse patient populations and across imaging hardware is not unified. External, multi‐institutional validation is rare in SHG‐specific work.

Furthermore, interpretability and explainability are essential for the clinical adoption of AI‐based SHG/PSHG classification. Clinicians, regulators and end users require transparency about how AI models make decisions, especially for imaging modalities that are unfamiliar (SHG) or have complex features. Studies outside of SHGs have shown that common explainable AI (XAI) methods often fail to meet clinical requirements, reliability, or truthfulness. For instance, ‘Evaluating Explainable AI on a Multi‐Modal Medical Imaging Task’ reported that many heatmap algorithms do not fulfil clinical expectations in correctly localising relevant features.^75^ Another review, ‘Guidelines and Evaluation of Clinical Explainable AI in Medical Image Analysis’, proposed specific criteria (e.g., understandability, clinical relevance, truthfulness) that are rarely addressed in existing AI imaging studies (Figure 6).^76^

Finally, regulatory and accreditation, this remains nontrivial. For AI tools to be used in clinical workflows, they must satisfy standards of safety, reproducibility, and performance across settings. In conclusion, while the near‐future adoption of AI‐based SHG workflows in clinical settings seems acceptable for high‐impact use cases (e.g., ex vivo cartilage assessment and surgical pathology), broader adoption will hinge on the assembly of larger, multicentre datasets, the embedding of explainable AI elements in model design, and the alignment of development with regulatory and accreditation frameworks.

## 5 FUTURE PERSPECTIVES OF COLLAGEN PATHOLOGY ASSESSMENT THROUGH SHG IMAGING

Advancements in the MNLO technique, computational strategies, and the integration of AI/ML for translational significance shape the future perspective of collagen pathology assessment through SHG and P‐SHG. To achieve millimetre depth penetration, the combination of SHG with deep tissue imaging, involving advanced tissue clearing and optimised preparation methods, is a promising area of research.^77^ These developments allow comprehensive three‐dimensional visualisation of collagen architecture compared with conventional thin‐section microscopy. This will reveal pathological remodelling at depths that were previously inaccessible to optical methods.^73^, ^78^ The ability of SHG to be intrinsic and label‐free remains a fundamental advantage, enabling the quantitative assessment of its arrangement and orientation without the variability introduced by histological staining. Linking the optical signature to biomechanical and pathological conditions by emerging approaches has the potential to yield more accurate diagnostic and prognostic biomarkers. The integration of AI/ML is expected to automate collagen quantification, enabling objective pathological assessment within digital pathology pipelines.

Researchers have made parallel efforts to miniaturise and reduce the cost of SHG imaging, driving its translation to medical‐grade devices. Multimodal imaging systems (2PF, 3PF, SHG, THG, and CARS) could offer comprehensive structural and functional tissue characterisation. In particular, SHG‐based fibre‐optic probes have demonstrated potential for in situ breast tumour detection, assessment of lymph node involvement, and real‐time biopsy guidance.^78^ Several start‐ups are actively developing compact, robust nonlinear‐imaging setups aimed at label‐free tissue assessment (e.g., Flash Pathology, TRANSVISTA, and Eleuthra Photonics). Beyond the technological horizon, SHG imaging is expected to impact a broader range of clinical research. Applications in oncology, fibrosis, cardiovascular pathology, etc. will benefit from deep‐tissue access and high‐resolution collagen mapping, offering novel insights into disease mechanisms and progression. Taken together, SHG/P‐SHG stands at a pivotal point: poised with great promise as a next‐generation, label‐free imaging modality but requires systematic efforts in standardisation, validation, and workflow integration before it can fulfil its translational potential in clinical practice.

## Supporting information



Supporting Information
