# Supplementary material for: Advancing collagen‐related pathology assessment through second‐harmonic generation imaging
Source: J Microsc. 2026 Apr 23;304(3):233–47. doi: 10.1111/jmi.70098 (PMC13740131; doi:10.1111/jmi.70098)
Supplement: Supplementary file 1 [file JMI-304-233-s001.docx]

**Advancing** **Collagen-Related Pathology Assessment through Second Harmonic Generation Imaging**

Jackson Rodrigues^1^, Manikanth Karnati^2^, Gagan Raju^2^, Edwin Pious^2^, Ankur Gogoi^3^, Guan-Yu Zhuo^1^, Nirmal Mazumder^2^, Fu-Jen Kao^1,^*

^1^Institute of Biophotonics, National Yang Ming Chiao Tung University, Taipei 11221, Taiwan

^2^Department of Biophysics, Manipal School of Life Sciences, Manipal Academy of Higher Education, Manipal, India

^3^Department of Physics, Jagannath Barooah University, Jorhat, Assam, 785001, India

*Corresponding Email ID: [fjkao@ym.edu.tw](mailto:fjkao@ym.edu.tw); fujenkao@gmail.com

1. **Theoretical framework of SHG & PSHG:**

As a nonlinear optical phenomenon, SHG occurs when two photons of the same frequency interact within a non-centrosymmetric material, producing a single photon of double the frequency (half the wavelength) of the incident photon. This process can be represented via Eqn. 1.

$\omega_{SHG}=2\omega$	(1)

where $\omega$ denotes the frequency of the incoming photon and where $\omega_{SHG}$ denotes the frequency of the SHG signal generated. More generally, in sum frequency generation (SFG), two photons of different frequencies ($\omega_{1}$ and $\omega_{2}$) combined to produce a photon at the sum frequency:$\omega_{SFG}=\omega_{1}+\omega_{2}$	(2)

SHG is therefore a special case of SFG in which $\omega_{1}=\omega_{2}=\omega$.

The nonlinear optical response behind the SHG signal can be described in terms of the electric field $E$ of the incident light and the polarization $P$, as given in Eqn. 3.

$P=\epsilon_{0}\left[ \chi^{\left( 1 \right)}E+\chi^{\left( 2 \right)}E^{2}+\chi^{\left( 3 \right)}E^{3}+\ldots\right]$	(3)

where $\chi{}^{\left( n \right)}$ represents the n^th^-order nonlinear susceptibility of the material, which affects the type of resulting nonlinear optical response. For example, $\chi{}^{\left( 1 \right)}$ governs linear optical responses such as refraction and absorption, whereas nonlinear optical processes arise from higher-order terms ($n>1$). SHG is a second-order susceptibility $\chi{}^{\left( 2 \right)}$ along with sum frequency generation (SFG) and difference frequency generation (DFG), whereas $\chi{}^{\left( 3 \right)}$ includes third-order processes such as third harmonic generation (THG), two-photon absorption, three-photon absorption, coherent anti-Stokes Raman scattering (CARS), and stimulated Raman scattering (SRS) ^1^.

Importantly, bulk centrosymmetric materials do not produce SHG since inversion symmetry requires $\chi{}^{\left( 2 \right)}=0$. Therefore, SHG arises only in media with broken symmetry (e.g., non-centrosymmetric crystals, interfaces, or biological fibrillar structures such as collagen). This is considered a selection rule in nonlinear optics for detecting the symmetry of materials.41 Finally, the efficiency of SHG depends on phase matching. The electric field associated with the incident laser beam induces second-order polarization in the bulk medium, which acts as a coherent source radiating at $2\omega$. For constructive interference across the propagation path, the generated second-harmonic wave must remain in phase with the fundamental wave. Under these conditions, contributions from different regions add coherently, leading to a strong second harmonic output. If these phases are not matched, destructive interference reduces the SHG output ^2^.

P-SHG imaging focuses on the changes in the SHG signal when the polarization states of the incident light are altered or when the polarization is analyzed as it passes through the sample. This process is generally referred to as polarization-in SHG (PI-SHG) and polarization-out SHG (PO-SHG). Polarization-in & polarization-out SHG (PIPO-SHG) is a technique that combines both the input and output light changes^3^. PI-SHG is one of the earliest renditions of P-SHG methods. A half-wave plate is placed in front of the sample to change the laser polarization. For a sample such as collagen, which has C6v symmetry, the angular dependence of the generated signal can be described by Eqn. (4).

$I_{2\omega}\propto\alpha_{0}+\alpha_{2}\cos2\left( \theta-\delta\right)+\alpha_{4}\cos4\left( \theta-\delta\right)$	(4)	where $\rho$ and $K$ represent the nonlinear susceptibility tensor components, *I* is the intensity of the SHG signal, $\omega$ is the frequency, and $\delta$ is the average orientation of the structure generating the SHG signal ^4^. In a circular dichroism PI-SHG (CD-SHG), instead of rotating linearly, left- and right-circularly polarized light are used as inputs. It is sensitive to the presence of chiral susceptibility components, out-of-plane tilt of the molecules, and phase differences between tensor elements. It is typically used when chiral structural information from a sample subjected to SHG imaging is needed.^5^

PO-SHG is usually coupled with Stokes-Mueller polarization SHG (SMP-SHG) to compute the degree of polarization (DOP), degree of circular polarization (DOCP), and degree of linear polarization (DOLP). It utilizes the information from the polarization state of the output SHG signal. Since PIPO SHG rotates both the input polarization (PSG) and output analyzer (PSA), it captures the SHG signal intensity for each combination of PSG and PSA angles. Although it results in more data and acquisition time, it increases the accuracy of fitting ρ & δ. By mathematically fitting polarization-resolved SHG data, values of ρ, δ, K, β, DOLP, etc., can be extracted that reveal how molecules are oriented, whether they are chiral, and how ordered they are.^6,7^

**References**^:^

1.	James DS, Campagnola PJ. Recent Advancements in Optical Harmonic Generation Microscopy: Applications and Perspectives. BME Front. 2021 Jan 25;2021.

2.	Mostaço-Guidolin L, Rosin N, Hackett TL. Imaging Collagen in Scar Tissue: Developments in Second Harmonic Generation Microscopy for Biomedical Applications. Int J Mol Sci. 2017 Aug 15;18(8):1772.

3.	Rouède D, Schaub E, Bellanger JJ, Ezan F, Tiaho F. Accordion-like collagen fibrils suggested by P-SHG image modeling : implication in liver fibrosis. 2018.

4.	Cisek R, Harvey M, Bennett E, Jeon H, Tokarz D. Polarization-Resolved SHG Microscopy for Biomedical Applications. In 2023. p. 215–57.

5.	Golaraei A, Kontenis L, Karunendiran A, Stewart BA, Barzda V. Dual‐ and single‐shot susceptibility ratio measurements with circular polarizations in second‐harmonic generation microscopy. J Biophotonics. 2020 Apr 5;13(4).

6.	Cisek R, Joseph A, Harvey M, Tokarz D. Polarization-Sensitive Second Harmonic Generation Microscopy for Investigations of Diseased Collagenous Tissues. Front Phys. 2021 Aug 30;9.

7.	Chen MC, Wang WH, Raju G, Mazumder N, Zhuo GY. Polarization-Resolved Second-Harmonic Generation for Tissue Imaging. In 2023. p. 259–304.
